# RETRACTION: The Mechanism of TDP‐43 Gene Expression on Inflammatory Factors and the JNK and p38 MAPK Signalling Pathways in Ischaemic Hypoxic Stress Dependence

**DOI:** 10.1111/iwj.70634

**Published:** 2025-04-23

## Abstract

**RETRACTION:** HuangH. , ZhangZ.F. , QinF.W. , TangW. , LiuD.H. , WuP.Y. , and JiaoF. , “The Mechanism of TDP‐43 Gene Expression on Inflammatory Factors and the JNK and p38 MAPK Signalling Pathways in Ischaemic Hypoxic Stress Dependence,” International Wound Journal 16, no. 3 (2019): 724–729, 10.1111/iwj.13087.30784197 PMC7949179

The above article, published online on 19 February 2019, in Wiley Online Library (http://onlinelibrary.wiley.com/), has been retracted by agreement between the journal Editor in Chief, Professor Keith Harding; and John Wiley & Sons Ltd. Following an investigation by the publisher, all parties have concluded that this article was accepted solely on the basis of a compromised peer review process. In addition, further investigation by the publisher found that the authors provided incomplete ethical approval information for animal experiments performed in the study. The editors have therefore decided to retract the article. The authors did not respond to our notice regarding the retraction.

**RETRACTION:** H. Huang , Z.F. Zhang , F.W. Qin , W. Tang , D.H. Liu , P.Y. Wu , and F. Jiao , “The Mechanism of TDP‐43 Gene Expression on Inflammatory Factors and the JNK and p38 MAPK Signalling Pathways in Ischaemic Hypoxic Stress Dependence,” International Wound Journal 16, no. 3 (2019): 724–729, 10.1111/iwj.13087.30784197 PMC7949179

The above article, published online on 19 February 2019, in Wiley Online Library (http://onlinelibrary.wiley.com/), has been retracted by agreement between the journal Editor in Chief, Professor Keith Harding; and John Wiley & Sons Ltd. Following an investigation by the publisher, all parties have concluded that this article was accepted solely on the basis of a compromised peer review process. In addition, further investigation by the publisher found that the authors provided incomplete ethical approval information for animal experiments performed in the study. The editors have therefore decided to retract the article. The authors did not respond to our notice regarding the retraction.

